# Correction to “Effects of short‐term tofogliflozin treatment on the insulin secretory capacity of people with type 2 diabetes: A randomized controlled trial, the TOP‐2ELM study”

**DOI:** 10.1111/jdi.70391

**Published:** 2026-07-13

Miyamoto M , Miya A , Nakamura A , Takahashi Y , Yamauchi Y , Yanagiya S , Takase T , Ono M , Takahashi K , Okada K , Kameda H , Cho KY , Atsumi T , TOP‐ELM Study Group . Effects of short‐term tofogliflozin treatment on the insulin secretory capacity of people with type 2 diabetes: A randomized controlled trial, the TOP‐ELM study. J Diabetes Investig 2026; 17: 627‐635. 10.1111/jdi.70245.PMC1304250341615833

In Table 4, the reported values for the change in the proinsulin/C‐peptide (PI/CPR) ratio were incorrect. The corrected values are shown below. This correction does not affect the statistical significance of the results or the conclusions of the article.

**Table 4:** Comparisons between the metformin and tofogliflozin groups with respect to glucose metabolism parameters at Week 4.

Variables	Mean change at Week 4				Change difference between groups *P* value
	Metformin group (*n* = 30)	*P* value	Tofogliflozin group (*n* = 35)	*P* value	
AUCglu	−2632 (−4313, −950)	0.0033	−3585 (−4554, −2617)	<0.0001	0.3028
AUCins	192 (−102, 638)	0.3911	−486 (−1064, −384)	<0.0001	0.0005
DI	0.005 (0.001, 0.008)	0.2972	0.007 (−0.001, 0.011)	0.1522	0.6451
ISSI‐2	0.001 (−0.089, 0.027)	0.3370	0.016 (−0.029, 0.086)	0.6883	0.1927
II	0.015 (0.002, 0.034)	0.1964	−0.003 (−0.058, 0.019)	0.4114	0.1972
MI	−0.39 (−1.01, 0.06)	0.0899	0.38 (−0.07, 0.94)	0.0854	0.0139
HOMA‐IR	−0.57 (−1.00, −0.15)	0.0307	−0.49 (−1.11, −0.38)	0.0009	0.6263
Proinsulin, pmol/L	−2.9 (−7.1, −0.6)	0.0083	−3.3 (−5.0, −1.9)	0.0025	0.7924
Proinsulin/CPR ratio	−1.1 (−1.9, −0.3)	0.0097	−1.5 (−2.3, −0.7)	0.0005	0.4572

Values are mean or median (95% confidence interval). Within‐group *P* values were obtained using the paired *t*‐test or the Wilcoxon signed rank test. The metformin and tofogliflozin groups were compared after 4 weeks using the unpaired *t*‐test or the Mann–Whitney *U*‐test.

Abbreviations: AUCglu, area under the curve for glucose; AUCins, area under the curve for insulin; CPR, C‐peptide; DI, disposition index; HOMA‐IR, homeostasis model assessment–insulin resistance; II, insulinogenic index; ISSI‐2, insulin secretion sensitivity index; MI, Matsuda index.

We apologize for this error.

